# MET-overexpressing myxofibrosarcoma frequently exhibit polysomy of chromosome 7 but not MET amplification, especially in high-grade cases: clinical and pathological review of 30 myxofibrosarcoma cases

**DOI:** 10.1186/s13000-018-0733-9

**Published:** 2018-08-21

**Authors:** Shirong Ma, Linni Fan, Yixiong Liu, Yingmei Wang, Kangjie Yu, Lifeng Wang, Na Fang, Fang Liu, Shuangping Guo, Zhe Wang

**Affiliations:** 1 State Key Laboratory of Cancer Biology, Department of Pathology, Xijing Hospital, Fourth Military Medical University, West Road #169, Xi’an, Changle, 710032 China; 2 Student Team 1, Class 3, Fourth Military Medical University, West Road #169, Xi’an, Changle, 710032 China; 3 Department of Pathology, Xinhua Hospital Affiliated to Shanghai Jiao Tong University School of Medicine, Kongjiang Road #1665, Shanghai, 200092 China; 4 Department of Pathology, Hubei Provincial Cancer hospital, Zhuodaoquan South Road #116, Wuhan, 430079 China; 5 Department of Pathology, Affiliated Foshan hospital, Sun Yet-sen University, Lingnan North Road#81, Foshan, 528000 China

**Keywords:** Myxofibrosarcoma, MET, FISH, Chromosome 7 polysomy

## Abstract

**Background:**

Myxofibrosarcoma (MFS) is one of the most common soft tissue sarcomas. Previous studies have shown that MET protein overexpressed in MFS patients and can serve as a prognostic factor. The reasons for MET protein overexpression include amplification of the MET gene, which is located on chromosome 7q. Triggered by an index case harboring chromosome 7 polysomy rather than MET gene amplification in myxofibrosarcoma, we investigated chromosome 7 polysomy in more cases.

**Methods:**

Immunohistochemistry and fluorescence in situ hybridization (FISH) were performed in 30 MFS cases (including 2 epithelioid variant) to detect the expression of MET protein and gene status.

**Results:**

MET was overexpressed in 14 cases out of 30, while thirteen cases were in higher FNCLCC grades (Grade 2–3). FISH showed that 11 cases having 3 signals on average of *Met* and more than 3 signals (Mean: 4.6) of centromere 7q (CEP7q). The MET/CEP7 ratio was about 0.65 on average, suggesting that chromosome 7 polysomy, rather than *Met* gene amplification, leading to the overexpression of MET protein in MFS. MET overexpression and chromosome 7 polysomy are positively correlated with higher Ki-67 index and higher grade and might have a high risk of local recurrence and metastasis.

**Conclusions:**

It might reveals another explain of MET overexpression in myxofibrosarcoma, providing a clue for the therapy of MFS.

## Background

Myxofibrosarcoma (MFS) is one of the most aggressive types of soft tissue neoplasm. It generally occurs in elderly patients and has a predilection for the limbs. Histologically, MFS features a multinodular growth pattern of spindle to polygonal sarcoma cells within variably myxoid stroma containing long curvilinear vessels [1]. Epithelioid MFS is a rare variant of MFS, accounting for < 3% of MFS but it is more aggressive than usual high-grade MFS, which has makes up approximately 70% of local recurrences and 50% of metastases [2]. In this way, the treatment of MFS is very tricky due to the limited diagnostic and prognostic factors.

MET, a member of the receptor tyrosine kinase family, is encoded by the *Met* gene on chromosome 7q31, has been found to be overexpressed in many cancers [3–6] and some soft tissue tumors [7–9], including MFS [10]. MET overexpression is usually correlated with tumor progression. Lee et al. found that MET overexpression independently predicts poor survival in MFS patients [10]. Regarding molecular mechanism, MET overexpression is considered attributable to amplification of the *Met* gene [3, 10–12]. Other studies have shown that polysomy of chromosome 7 can account for the overexpression of MET in chordomas [9] and primary colorectal cancer [13]. In this way, it is not clear whether there is any polysomy in chromosome 7 in MFS. There is also no information available concerning MET expression and gene status in epithelioid variant MFS. In order to further confirm the relationship between *MET* and MFS, especially the epithelioid variant, we used immunohistochemistry and FISH to detect the MET expression and gene status in 30 MFS, including 2 epithelioid variants, and reviewed clinical and pathologic features as well as clinical outcomes.

## Methods

### Patients’ data

**Fig. 1:**
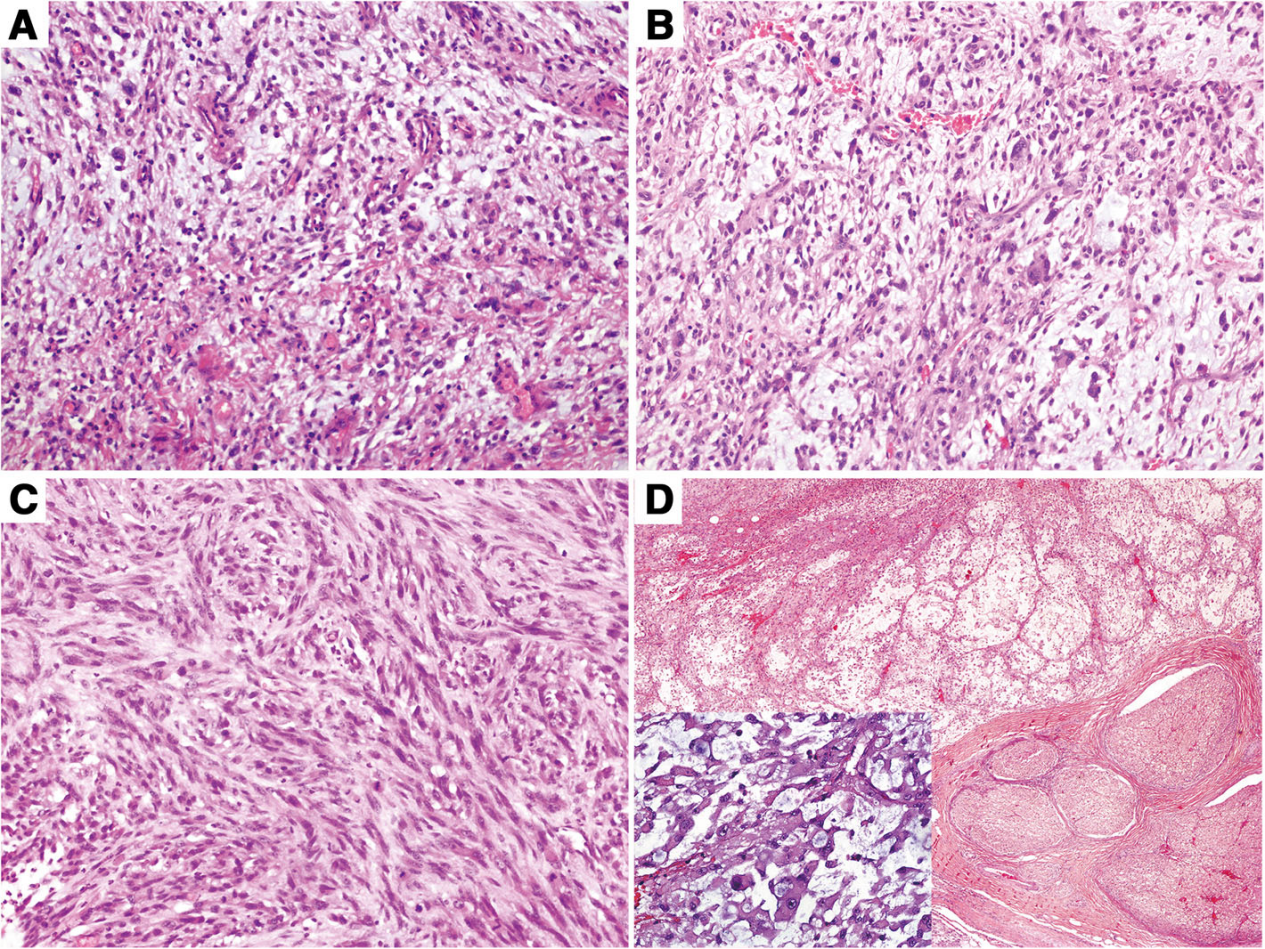
Mophology of MFS: Spindle to polygonal sarcoma cells within variably myxoid stroma containing long curvilinear vessels. **a**: FNCLCC Grade 1 MFS; **b**: FNCLCC Grade 2 MFS; **c**: FNCLCC Grade 3 MFS; **d**: Epithelioid variant of MFS, tumor cells is an infiltrative multinodular growth pattern, with alternation of hypercellular and hypocellular myxoid areas. Tumor cells had epithelioid morphology with abundant eosinophilic cytoplasm and oval or plump spindle nuclei, scattered in the myxoid background (inset).

A total of 30 patients were identified and retrieved from several pathological centers: Xijing Hospital (12 cases, including 1 epithelioid subtype), Xihua Hospital (1 case, epithelioid subtype), Hubei Provincial Cancer Hospital (11 cases), Affiliated Foshan Hospital, Sun Yet-sen University (6 cases). The 30 patients included 16 males and 14 females with a mean age of 56 years (range: 21–87 years) (Table 1). All tumors were graded according to the FNCLCC: 6 cases were classified as grade 1 (Fig. 1a), and 12 cases as grade 2 (Fig. 1b) and the left 12 cases as grade 3 (Fig. 1c). Two cases of grade 3 were epithelioid MFS, showing an infiltrative multinodular growth pattern with alternation of hypercellular and hypocellular myxoid areas. Tumor cells had epithelioid morphology with abundant eosinophilic cytoplasm and oval or plump spindle nuclei, scattered in the myxoid background (Fig. 1d). Here, 15 of the 30 cases were followed up for 7 to 42 months. All the slides were re-examined by two pathologists to ensure correct diagnosis. The specimen collection and study procedures were approved by the Ethics Committee of Xijing Hospital.

**Table 1:** Clinicopathological characteristics of 30 MFS cases

Case	Age (≥56)	Gender	Location	Tumor size(cm)	FNCLCC grade	MET expression	chromosome 7 polysomy	Ki67 Index	Follow-up
1	Y	F	Left leg	4.5	Grade 2	+	+	40%	local recurrence
2	N	M	Left ischium	8	Grade 2	–	–	20%	NED
3	Y	M	Right shoulder	2.2	Grade 2	–	–	15%	NED
4	Y	F	Left leg	1.8	Grade 1	–	–	15%	NED
5	Y	F	Right shoulder	5	Grade 3	+	+	45%	local recurrence
6	N	M	Right leg	1	Grade 2	–	–	15%	seven months later, die of metastasis
7	N	M	Back	3.5	Grade 3	+	+	30%	local recurrence
8	N	F	Left leg	3	Grade 1	–	–	3%	NED
9	Y	F	Left scapula	11	Grade 3	+	+	40%	local recurrence and metastasis to axillary
10	N	M	Right forearm	4	Grade 1	–	–	5%	NED
11	Y	F	Right leg	1.5	Grade 1	–	–	3%	NED
12*	N	M	Right foot	1.8	Grade 1	–	–	6%	NED
13*	N	F	Ankle	4	Grade 2	+	+	15%	N/D
14	N	F	Left leg	21	Grade 2	–	–	40%	N/D
15	Y	M	Right armpit	4.5	Grade 2	–	–	40%	N/D
16	N	F	Left arm	1.8	Grade 1	+	+	10%	N/D
17	Y	M	Left shoulder	4	Grade 2	+	–	80%	N/D
18	Y	M	Right arm	4	Grade 3	+	+	70%	N/D
19	Y	F	Right leg	19	Grade 3	+	+	20%	N/D
20	N	M	Left leg	7.5	Grade 3	+	–	70%	N/D
21	Y	M	Right chest wall	19	Grade 2	–	–	60%	N/D
22	N	M	Right chest wall	1.5	Grade 2	–	–	60%	N/D
23	Y	M	Left arm	11	Grade 3	+	+	70%	local recurrence
24	Y	M	Left leg	13	Grade 3	–	–	60%	N/D
25	Y	M	Right arm	6	Grade 3	+	+	40%	N/D
26	N	F	Left shoulder	11	Grade 3	–	–	50%	local recurrence
27	Y	F	Right leg	9	Grade 3	+	+	20%	N/D
28	Y	F	Right leg	9	Grade 2	–	–	60%	N/D
29	Y	M	Left leg	2	Grade 2	–	–	40%	local recurrence
30	Y	F	Neck	3.5	Grade 3	+	–	40%	N/D

*: epithelioid variant *NED* no evidence of disease. *N/D* not done

### MET immunohistochemistry

Paraffin-embedded sections 4 μm in thickness were deparaffinized and treated with 3% (*v*/v) hydrogen peroxide to block endogenous peroxidase activity. They were heated in a microwave for antigen retrieval in 0.01 M citrate buffer at pH 6.0 for 15 min, followed by incubation in 10% normal goat serum for 30 min to block the nonspecific antibody-binding sites. The sections were then incubated overnight at 4 °C with a primary antibody against total MET (1:100, EP1454Y, Abcam). Then a standard rapid EnVision technique (Dako, Glostrup, Denmark) was used to detect the protein conjugates and develop the color. Finally, the sections were counterstained with hematoxylin and visualized. Serial sections were run in parallel with serum in which the primary antibody had been replaced with PBS and rabbit IgG as blank and negative controls, respectively.

MET reactivity was predominantly seen in the cytoplasm and membrane. Immunostaining was evaluated on the basis of the staining intensity (negative, weak, moderate, strong) and the percentage of cells stained, according to the previous studies of MET IHC scoring criteria [11, 14] as follows: 0 (no staining or < 50% of tumor cells with any intensity), 1+ (≥ 50% of tumor cells with weak or higher staining but < 50% with moderate or higher intensity), 2+ (≥ 50% of tumor cells with moderate or higher staining but < 50% with strong intensity), 3+ (≥ 50% of tumor cells stained with strong intensity), MET overexpression was defined as a score of 2+ or 3+. IHC scores were independently evaluated by two pathologists who were blinded to all the cases.

### MET fluorescence in situ hybridization

FISH was performed on formalin-fixed, paraffin-embedded tissue sections in accordance with the manufacturer’s instructions. Unstained 4 μm sections were placed on electrostatically charged slides and then evaluated using MET (7q31) probe and centromere 7q (CEP7q) dual-color probe (LBP, Guangzhou, China). The hybridized slides were reviewed on an Olympus IX-50 microscope (Olympus, Tokyo, Japan) at 100× magnification with oil immersion using a DAPI/Green/Red triple band pass filter set. The tissues were scored by evaluating a minimum of 40 tumor nuclei per sample. The amplification of MET was defined as 2 ≦ MET gene (red)/CEP7q (green) per cell on average. We defined polysomy of chromosome 7 as three or more signals on average of CEP7 (green) per cell [9, 13]. To avoid false-positive results originating from the nuclear truncation that occurs in a subset of cells in paraffin-embedded samples, we excluded overlapping cells that did not clearly have separate nuclei from the current analysis.

### Statistics

Statistical analyses were performed using the Statistical Program for Social Sciences (SPSS) software (Version 17.0, SPSS Inc., Chicago, IL, U.S.). Chi-square tests were used to assess the differences and correlations between the expression of MET and the clinicopathological features of patients. Both univariate and multivariate survival analyses were performed. The survival analysis was performed using the Kaplan-Meier method. All tests were two-sided. *P*-values < 0.05 were considered significant.

## Results

### MET overexpression and chromosome 7 polysomy are preferentially detected in higher FNCLCC grade MFS and the epithelioid variant

**Fig. 2:**
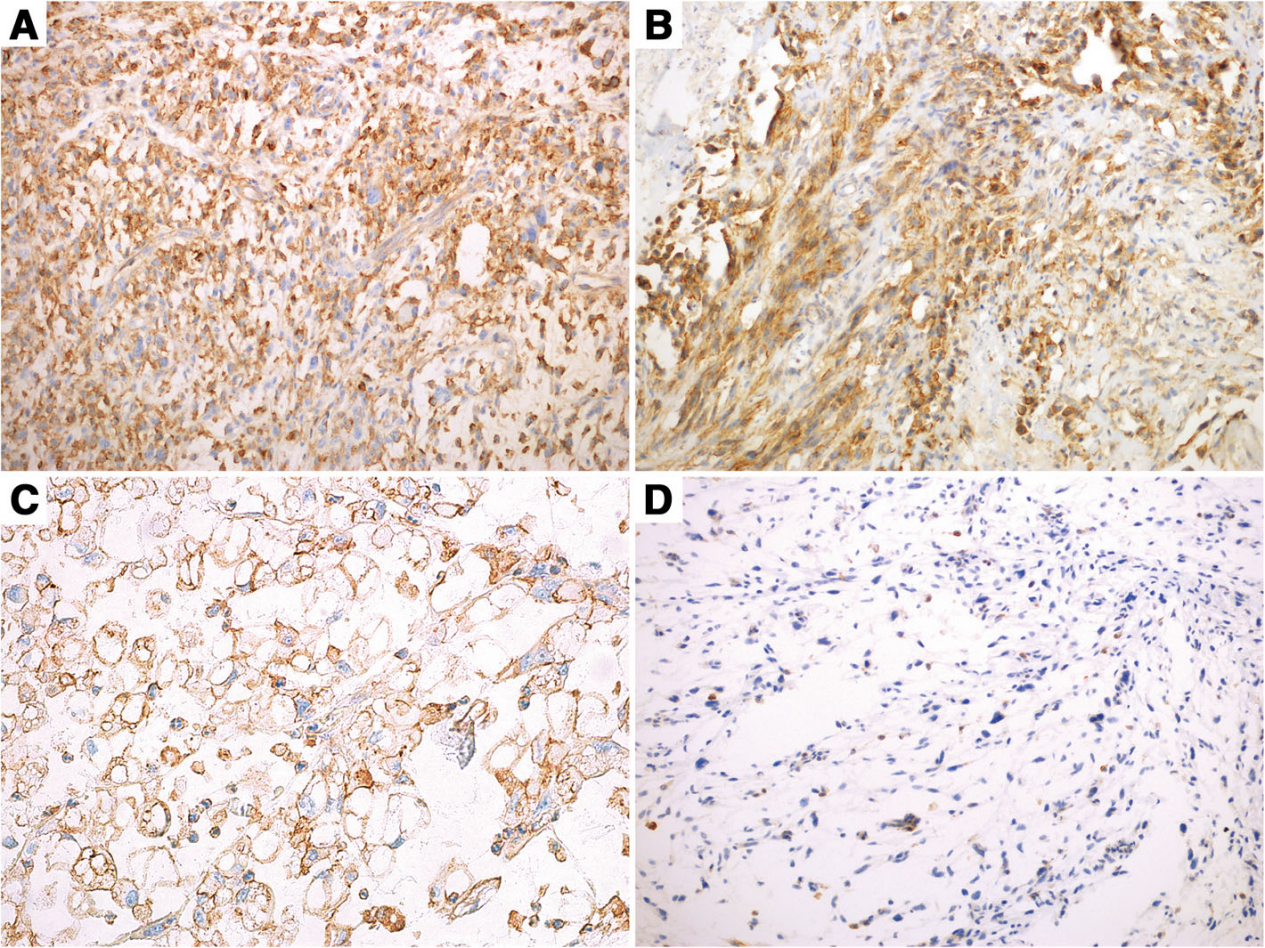
MET immunostain shows positive cytoplasm staining in the FNCLCC Grade 2 (**a**), Grade 3 (**b**), and Epthelioid MFS (**c**), while negative expression in FNCLCC Grade 1 MFS (**d**)

For immunohistochemistry, MET overexpression was observed in 14 cases (including 1 case of epithelioid variant) of 30 cases (46.7%, Table 1), which consisted of 6 males and 8 females with a median age of 61 years (range, 38–87 years). Moreover, the positivity varied with tumor grade, and the positive cases were 1, 3 and 10 cases for grade 1 to 3, respectively (Fig. 2a, b), one of which was high grade epithelioid myxofibrosarcoma (Fig. 2c). Other MET-negative cases were of lower grade (Fig. 2d).

**Fig. 3:**
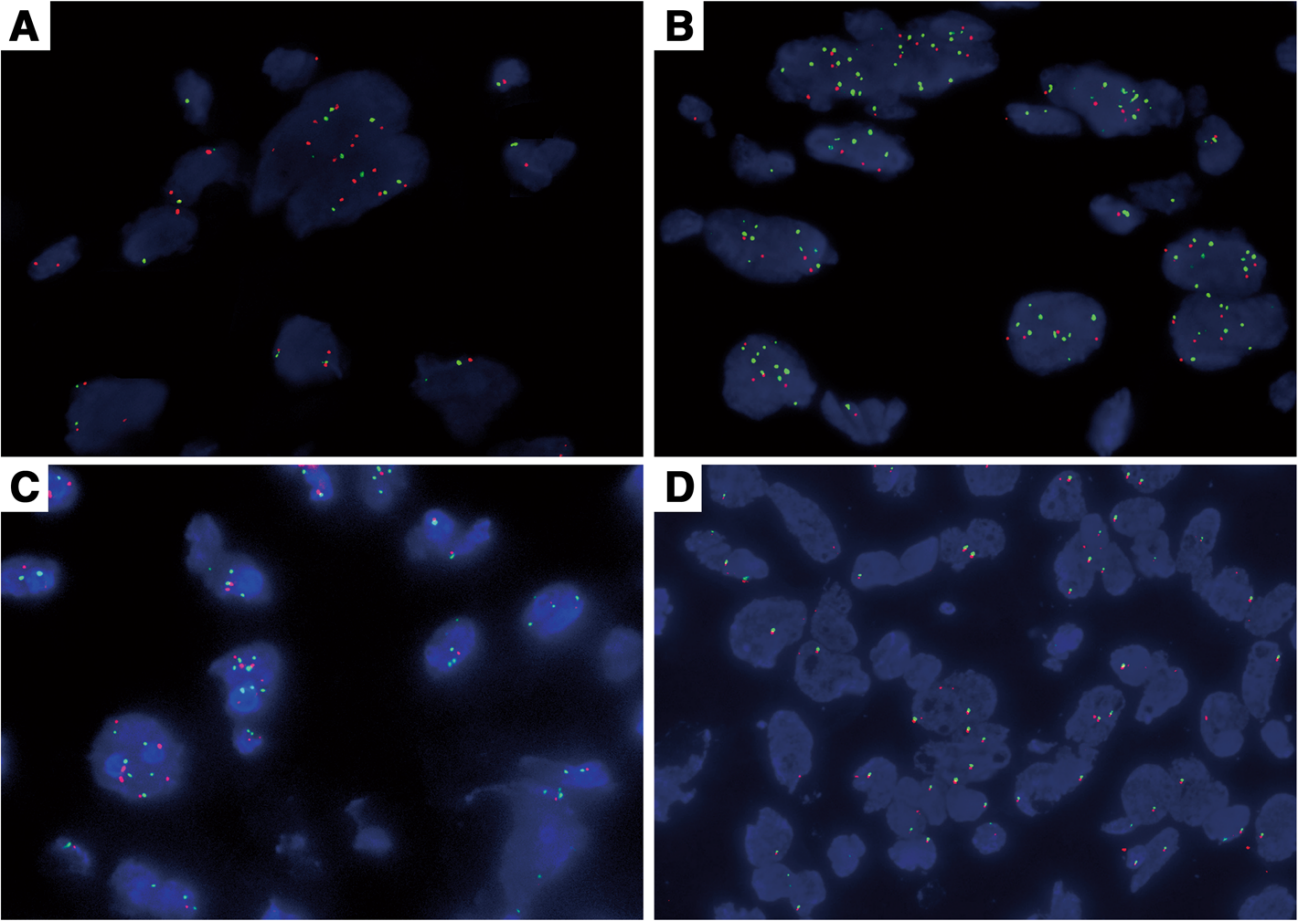
FISH examination of met gene status by *MET* (red)/CEP 7(green). Chromosome 7 polysomy, rather than *MET* amplification, was found in FNCLCC Grade 2 cases (**a**), Grade 3 cases (**b**), and epithelioid variant (**c**). Lower FNCLCC grade cases are usually negative for either *MET* amplification or chromosome 7 polysomy (**d**)

We further examined *MET* gene status using FISH with the probe MET/CEP7. There were also 11 cases with 3 signals on average of MET and more than 3 signals (4.6 signals on average) of CEP7. However, the MET/CEP7 was about 0.65 on average. This is suggested that chromosome 7 polysomy, rather than MET amplification, led to the overexpression of MET protein. Among the 11 cases, chromosome 7 polysomy was preferentially detected in cases with higher FNCLCC grade, for 2 of the 11 cases that were classified as grade 2 (Fig. 3a), and the other 8 cases that were classified as grade 3 (Fig. 3b), including one epithelioid variant (Fig. 3c). In contrast, most of the negative cases had lower FNCLCC grade (Fig. 3d).

### MET overexpression and chromosome 7 polysomy are positively correlated with higher FNCLCC grade and higher Ki-67 index.

As shown in Table 2 and Figs. 2 and 3, MET overexpression and chromosome 7 polysomy were statistically positively correlated with higher FNCLCC grade (*P =* 0.004, *P =* 0.021). In contrast, MET overexpression and chromosome 7 polysomy were almost absent from grade 1 tumors in our study. The 11 positive cases were also positively correlated with higher mitotic rate (*P* = 0.029), but it was not related to age, gender, or location.

**Table 2:** Clinicopathological characteristics and associations with MET immunoexpression and chrosome 7 polysomy in 30 MFS

Parameters	C-MET expression		*P*	(CEP7 polysomy)		*P*
	+	–		+	–	
Age			0.232			0.757
≥ 56	10	8		7	11	
<56	4	8		4	8	
Gender			0.282			0.156
Male	6	10		4	12	
Female	8	6		7	7	
Tumor size			0.812			0.892
≥ 6.7	5	6		4	7	
<6.7	9	9		7	11	
Location			0.765			0.757
limbs	8	10		7	11	
other	6	6		4	8	
FNCLCC grade			*0.004*			0.021*
Grade 1	1	5		1	5	
Grade 2	3	9		2	10	
Grade 3	10	2		8	4	
Ki-67 Index			*0.011*			0.029*
≥ 0.33	10	4		8	6	
<0.33	4	12		3	13	
Local recurrence and metastasis^a^			*0.010*			0.010*
Yes	5	3		5	3	
No	0	7		0	7	

*p<0.05 is considerably significantlly. ^a^:*n* = 15, half of the cases were censored

### Clinical outcome

Except for one censored case, 15 patients were followed up for survival. One patient died of metastasis seven months after surgery. There were 7 cases had local recurrence, of which one had metastasis to axillary (Table 1). Among the 15 follow up cases, there were 5 cases positive for MET overexpression and chromosome 7 polysomy, and this correlation was statistically significant (*P* = 0.010), which indicated that MFS patients with MET overexpression or chromosome 7 polysomy might have a high risk of local recurrence and metastasis. However, *Log-rank* test showed there to be no significant relationship between MET overexpression and chromosome 7 polysomy patients and the survival time of MFS patients after surgery (*P* = 0.317).

## Discussion

MFS is one of the most aggressive types of soft tissue neoplasm. MFS generally occur in elderly patients and has a predilection for the limbs. Clinically, MFS is tend to recur persistently, even after wide resection [15–18]. According to the previous studies, local recurrence rates of MFS have ranged from 22 to 79%. Moreover, about one third of locally recurrent MFS cases could progress to a higher grade, and 20–35% of the high grade neoplasm may develop hematogenous metastases [19–21]. In our study, 18 out of 30 patients (60%) had the tumors in their limbs. For followed-up study**,** 8 out of 15 cases (53.3%) had recurrence and 2 cases had metastasis. Moreover, one patient died of metastasis seven months after surgery. All 8 recurrent cases were of higher FNCLCC grade (grade 2–3). All these findings were consistent with previous studies. However, the mean age of the patients in our study was 56 years, which was younger than that of previous studies 60 years.

There have been relatively few studies of cytogenetic and molecular genetics of MFS. Willems et al. proposed the concept of progression of MFS as a multistep genetic process ruled by genetic instability. It has been reported that MFS harbors complex karyotypes but without specific structural aberrations. Chromosome 7 is involved in the only recurrent gain [22, 23]. The *Met* gene, mapped to chromosome 7q, encodes a proto-oncogene, regulating both cell motility and cell growth to allow stem cells and progenitor cells to grow invasively [24]. The gene has been shown to be over-expressed in many tumors, including ovarian [25], lung [26], gastric [27], and colon cancers [13]. Its overexpression usually correlated with a poor prognosis [10].

In our study, immunohistochemical results indicated that MET was over-expressed in 14 out of 30 MFS cases, especially in cases with higher FNCLCC grades and the epithelioid variant (*P* = 0.004). MET overexpression is also positively correlated with higher Ki-67 index (*P* = 0.011). However, there was no relationship with age, gender, nor tumor location in MFS patients. Also, MET overexpression usually predicted high risk of local recurrence and metastasis (*P* = 0.010). These results were consistent with those reported by Lee [10], which showed that MET is expressed in most MFS cases and its overexpression is closely related to deep location, higher grade, and more advanced stage. However, we did not find any prognostic implication of MET due to the limited follow-up data.

*Met*, also known as hepatocyte growth factor receptor (HGFR), is reported to be a tyrosine kinase receptor in epithelial cells. Through combination with HGF, Met could activate RAS-MAPK or PI3K-Akt signaling pathway to promote cell motility and proliferation [28]. MET is deregulated in many types of human malignancies, including cancers of the kidney, liver, stomach, breast, brain, bone, and blood. Also, abnormal MET activation in cancer correlates with poor prognosis, where aberrantly active MET triggers tumor growth, angiogenesis and metastasis. Due to its oncogenic features, several MET inhibitors have been used to treat cancers in clinical trials. Oral S49076, a unique MET/AXL/FGFR inhibitor, has been used in advanced solid tumors. Another MET inhibitor Crizotinib could prevent peritoneal dissemination in pancreatic cancer. Thus, assessment of the expression and gene status of MET, could be helpful for the potential target therapy for MFS patients.

In previous studies, MET overexpression is considered the result of *MET* gene amplification. For this reason, we also tested the *MET gene* status by FISH with the probe MET/CEP7, and the results showed that five cases which have 3 signals on average of *MET* but more than 3 signals (4.6 signals on average) of CEP7. The MET/CEP7 was about 0.65 on average, suggesting that chromosome 7 polysomy, rather than *MET* gene amplification, led to overexpression of MET protein in MFS. However, the mechanisms underlying the action of chromosome 7 polysomy in MFS need further study.

## Conclusions

Our work confirmed that MET overexpression was more frequently found in high grade MFS and the epithelioid variant. Chromosome 7 polysomy, rather than *MET* gene regional amplification, might account for the overexpression of MET protein in MFS, which could shed a light on the therapies for MFS.

## Abbreviations

CEP7: centromere 7q
FISH: fluorescence in situ hybridization
HGFR: hepatocyte growth factor receptor
MFS: myxofibrosarcoma
